# Surgical Outcomes Following Patella Fracture Repair: A Single-Center Retrospective Cohort Study

**DOI:** 10.7759/cureus.92343

**Published:** 2025-09-15

**Authors:** Jacob R Feathers, David Fellows, Edward Richardson, Mohammed Khatir, Akhshay George, Neil Ashwood

**Affiliations:** 1 Trauma and Orthopedics, Cwm Taf Morgannwg University Health Board, Pontypridd, GBR; 2 Trauma and Orthopedics, Oxford University Hospitals NHS Foundation Trust, Oxford, GBR; 3 Trauma and Orthopedics, Gloucestershire Hospitals NHS Foundation Trust, Gloucester, GBR; 4 Orthopedics, University Hospitals of Derby and Burton NHS Foundation Trust, Burton, GBR; 5 Trauma and Orthopedics, University Hospitals of Derby and Burton NHS Foundation Trust, Derby, GBR

**Keywords:** acute trauma care, open reduction of patella, patella fracture, patella surgery, tension band wire

## Abstract

Introduction

Patella fractures are an uncommon yet complex injury, accounting for approximately 1% of presentations to orthopedic departments. Surgical management is indicated for unstable, displaced, or comminuted fracture patterns, with the aim of restoring the extensor mechanism and preserving knee function. Various surgical techniques are available, each with differing complication profiles. This study evaluated surgical outcomes following patella fractures over a 15-year period within a single-center orthopedic department.

Methods

A retrospective cohort study was conducted. A single-center trauma and orthopedic database was reviewed over a 15-year period from 2008 to 2023. Inclusion criteria comprised patients presenting with patella fractures. Exclusion criteria included patients transferred to another healthcare provider for management, those lost to follow-up, or patients who did not engage with planned follow-up. Data analyzed included fracture morphology, time to surgery, surgical technique, complications, reoperation rates, and recovery duration. All patients followed a standardized physiotherapy protocol.

Results

A total of 100 patella fractures were included, of which 65/100 (65%) underwent surgical fixation. The mean age of patients requiring operative management was 60 years (range: 17-95), and 64.6% were female. The most prevalent fracture morphologies were transverse (44.6%) and comminuted (38.4%). The most commonly used surgical technique was tension band wiring (TBW, 64.6%), followed by cerclage wiring, partial excision with tendon repair, and cannulated screws. Across all surgical patients, the average time to recovery was 7.4 months. TBW had a mean recovery duration of 6.8 months. The shortest recovery times were observed for open reduction and internal fixation with screws (3.25 months), and the longest with excision of distal bone (24 months). Patients managed conservatively had the shortest recovery time at 2.7 months. Postoperative complications occurred in 44.6% of surgical patients. The leading complication was metalwork irritation (24.6%), which led to reoperation in 13/65 patients. Other complications included malunion, stiffness, wound infection, and non-union. There was no significant difference in recovery time between patients operated on within one day versus later.

Conclusions

Surgical management of patella fractures is associated with good functional outcomes; however, this study found a high rate of postoperative complications and subsequent reoperations. The most predominant reason was hardware irritation associated with TBW. These findings highlight the need for careful planning and individualization based on fracture morphology and patient factors. Recovery time is influenced by the fixation method, and current evidence on optimal postoperative recovery expectations is limited. These results underscore the need for further research to guide surgical decision-making and rehabilitation protocols.

## Introduction

The patella plays a crucial role in knee biomechanics [1]. It is the largest sesamoid bone in the body and is encased by the quadriceps tendon. The patella increases the efficiency of force transmission across the knee by enhancing the leverage of the quadriceps during knee extension [2]. Located superficially within the knee, it is subject to high mechanical stresses and is therefore susceptible to both direct and indirect trauma [3]. Fractures can result from direct impact to the knee, high-energy injuries, or avulsion from the quadriceps tendon. Patella fractures account for approximately 1% of injuries seen in orthopedic departments, affecting roughly 15 per 100,000 people [4,5].

Patella fractures can be classified based on radiological morphology. Transverse fractures are the most common, accounting for 56% of injuries [6], and typically result from sudden, forceful contraction of the quadriceps against a flexed knee. Comminuted fractures usually occur due to a direct blow or high-energy trauma and are often associated with soft tissue injury or disruption of the extensor mechanism [7]. Vertical fractures are less common, accounting for approximately 22% of patella fractures, and generally arise from direct compression of the patella on a hyperflexed knee [8].

Conservative management of patella fractures is reserved for non-displaced or minimally displaced fractures (<2 mm articular step-off) without disruption of the extensor mechanism [9] or for patients deemed unfit for surgery. Surgical fixation is typically required for fractures that are displaced, comminuted, unstable, or associated with extensor mechanism disruption.

Tension band wiring (TBW) has long been considered the gold standard for the most common transverse fracture pattern. TBW employs wires in a figure-eight configuration to convert tensile forces during knee flexion into compressive forces at the fracture site [10]. However, TBW may not be appropriate for certain oblique, vertical, or comminuted fracture orientations. TBW is also associated with complications such as metalwork irritation or failure, which may necessitate hardware removal [11].

Oblique fractures may not be amenable to TBW when the fracture line is not perpendicular to the tensile force. In such cases, alternative internal fixation methods, such as locking plates or cannulated screws, may be required. Comminuted fractures can be particularly challenging to fix, and in severe cases, partial or total patellectomy may be necessary [12].

Surgeons must therefore carefully consider the fracture pattern, patient comorbidities, and the biomechanics of fixation when planning management. This study aims to assess the outcomes of patients undergoing surgical management of patella fractures.

## Materials and methods

Study design

This study was a retrospective observational cohort study examining patella fractures. The primary aim was to analyze the choice of surgical fixation for patella fractures, along with postoperative complications and reoperation rates.

Setting

Data were obtained from a single-center trauma and orthopedic department in the United Kingdom. Both primary surgery and subsequent follow-up were conducted within the same trust. Postoperatively, patients were followed up in the same department, with outcomes, complications, and any further operations documented in the clinical notes.

Duration

This study was conducted over a 16-year retrospective period, from 2007 to 2023.

Patient recruitment

Patients were identified from the trauma and orthopedic department registry, which records admissions and treatments for patella fractures during the study period. Eligibility was determined by retrospectively reviewing clinical records and cross-referencing them with the inclusion and exclusion criteria.

Inclusion criteria 

All patients aged 16 years or older presenting with a patella fracture requiring treatment were included. To minimize selection bias, all eligible patients within the study period were initially included.

Exclusion criteria

Patients transferred to another healthcare provider (trust) for primary surgery, those lost to follow-up, or patients who did not engage with planned follow-up were excluded.

Data collection

Data were sourced from the trauma and orthopedic department registry, and variables collected included preoperative factors such as fracture morphology, patient demographics, and time to surgery; intraoperative details, including the surgical technique used and any intraoperative complications; and postoperative outcomes, including time to recovery (defined as fracture healed and discharged from clinic), complications, and reoperation rates. Patient electronic and paper records were reviewed to obtain these data, which were anonymized and analyzed independently from the operating surgeons.

Statistical analysis

Descriptive statistics were used to summarize patient characteristics and surgical outcomes. Comparative analyses (e.g., stratification by surgical technique) were limited due to cohort heterogeneity and sample size constraints. Data analysis was performed using Microsoft Excel (Microsoft Corporation, Redmond, WA, USA).

Ethics

This study was registered as a clinical audit (audit ID: UHDBS542) within the health board’s governance framework. Ethical approval was granted via the clinical audit pathway, and patient consent was waived due to the retrospective nature of the study and anonymized data handling.

## Results

This study identified 106 patients with patella fractures. Four patients were excluded because they were transferred to another hospital, and a further two patients were excluded due to loss to follow-up. Of the remaining 100 patients, 65 (65%) underwent surgical fixation. Among those undergoing surgery, 23/65 (35.3%) were male and 42/65 (64.6%) were female. The average age of patients was 60 years (median 66, range 17-95). Laterality of the fractures showed that 29/65 (44.6%) were left-sided and 36/65 (55.4%) were right-sided. These data are summarized in Table 1.

**Table 1:** Patient demographics This table summarizes the demographics of patients included in our study, showing both the number of patients (n) and associated percentages. It details the included and excluded patients, the distribution of surgical versus conservative management, patient gender, age, and fracture laterality.

Category	N (%)
Total patients with patella fractures identified	106
Included in the study	100
Patients undergoing surgical fixation	65 (65%)
Conservatively managed	35 (35%)
Sex (entire study group)	
Male	32 (32%)
Female	68 (68%)
Sex (surgical fixation group)	
Male	23 (35.3%)
Female	42 (64.6%)
Age (entire study group)	
Mean	60 years
Median	66 years
Range	17-95 years
Laterality (surgical fixation group)	
Left	29 (44.6%)
Right	36 (55.4%)

**Figure 1:**
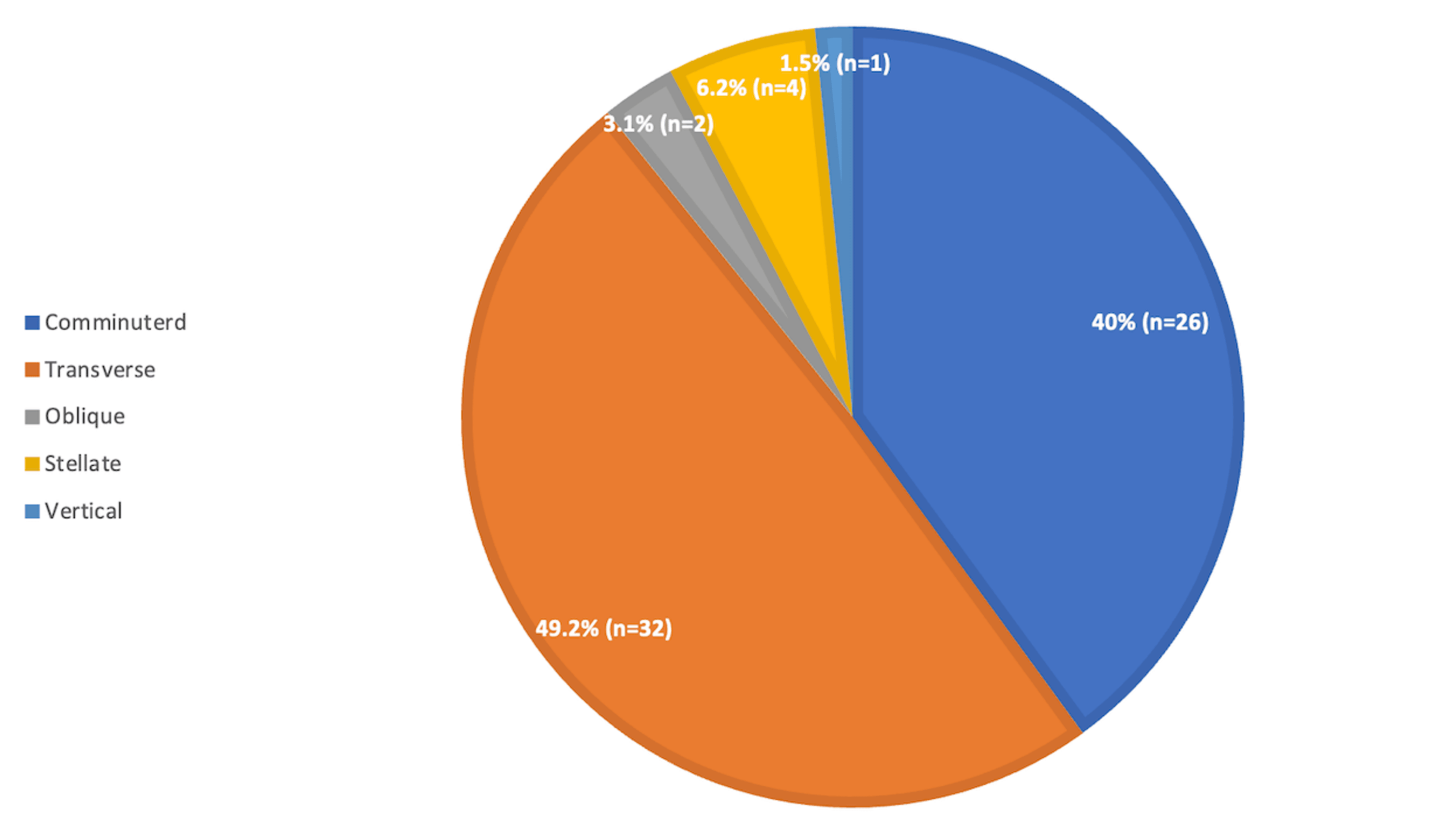
Frequency of patella fracture morphology requiring surgical fixation This figure illustrates the distribution of patella fracture morphologies among patients requiring surgical fixation. Each segment represents the percentage of patients along with the corresponding n count. Fracture patterns included comminuted, transverse, oblique, stellate, and vertical.

The distribution of fracture patterns among patients undergoing surgical fixation was as follows: 26/65 (40%) comminuted, 32/65 (49.2%) transverse, 2/65 (3.1%) oblique, 4/65 (6.2%) stellate, and 1/65 (1.5%) vertical fractures (Figure 1).

Regarding surgical techniques, 42/65 (64.6%) patients underwent TBW, while 4/65 underwent tension band suturing. Five patients (5/65) underwent partial excision with patella tendon repair, 2/65 underwent star plating, and 2/65 underwent open reduction and internal fixation (ORIF) with screws. Seven patients (7/65) underwent ORIF with cerclage wires or sutures, 2/65 underwent fiber tape ORIF, and 1/65 underwent excision of distal bone. These data are summarized in Table 2.

**Table 2:** Number of surgical procedures undertaken during the study This table summarizes the number of surgical procedures performed on patients presenting with patella fractures to our department. ORIF, open reduction and internal fixation; TBS, tension band suturing; TBW, tension band wiring

Type of surgery	Number of patients
TBW ORIF	42
TBS	4
Partial excision + patella tendon repair	5
Star plate	2
ORIF with screws	2
ORIF with cerclage wire/suture	7
Fiber tape ORIF	2
Excision of the distal bone	1

The average time to surgery and time to recovery are shown in Table 3. Fiber tape fixation had the shortest time to surgery, averaging one day, with a relatively good recovery time of 4.75 months. ORIF with cerclage wires was the next fastest to surgery at 1.14 days but had a prolonged recovery time of 13.7 months. ORIF with screws had the quickest recovery at 3.25 months, while excision of the distal bone was associated with the longest recovery at 24 months.

**Table 3:** Average time to surgery (days) and time to recovery (months) by type of surgery This table summarizes the average duration to surgery (in days) for each surgical technique, along with the average time to recovery (in months). ORIF, open reduction and internal fixation; TBS, tension band suturing; TBW, tension band wiring

Surgery type	Average days to surgery	Average months to recovery
Fiber tape ORIF	1	4.75
TBW	2.5	6.8
TBS	5.75	4.25
Partial excision + patella tendon repair	10.4	5.35
Star plate	10.5	8
ORIF with cerclage wires	1.14	13.7
ORIF with screws	2.5	3.25
Excision of the distal bone	3	24

A total of 27 patients (41.5%) underwent surgery within one day of hospital presentation, with an average recovery time of 7.53 months. The remaining 38 patients (58.5%) had surgery two days or longer after presentation, with an average recovery of 7.36 months. Among the 35 patients managed conservatively, the average time to recovery was 2.69 months.

Of the conservatively managed patients, 3/35 (8.6%) experienced complications, including two malunions and one case of stiffness. Postoperative complications for all surgically managed patients are summarized in Table 4. During the follow-up period, 29/65 (44.6%) patients developed a postoperative complication across all surgical techniques. The predominant complication was metalwork irritation, occurring in 16 cases. Metalwork removal was required in 13/65 cases, with 11 following TBW and two following cerclage wiring. One patient undergoing TBW required metalwork trimming, partial patellectomy, and extensor mechanism repair.

**Table 4:** Postoperative complications by surgical technique This table presents the numerical count of postoperative complications associated with each surgical technique. ORIF, open reduction and internal fixation; TBS, tension band suturing; TBW, tension band wiring

Postoperative complication	Total	TBW	ORIF cerclage wire/suture	ORIF star plate	ORIF screws	Fiber tape ORIF	Partial excision + tendon repair	Excision distal bone	TBS
None	36	23	1	1	1	1	5	0	4
Metalwork irritation	16	12	3	1	0	0	0	0	0
Cast pressure sore	2	2	0	0	0	0	0	0	0
Pulmonary embolism	1	1	0	0	0	0	0	0	0
Delayed union	2	2	0	0	0	0	0	0	0
Malunion	4	3	0	0	0	1	0	0	0
Partial loss of reduction	1	1	0	0	0	0	0	0	0
Implant failure	1	1	0	0	0	0	0	0	0
Nonunion	1	0	1	0	0	0	0	0	0
Stiffness	5	1	2	1	1	0	0	0	0
Osteoarthritis	2	2	0	0	0	0	0	0	0
Anterior knee pain	2	0	2	0	0	0	0	0	0
Infection	3	2	1	0	0	0	0	0	0
Tendinopathy	1	0	0	0	0	0	0	1	0

## Discussion

Over the follow-up period, 36/65 (55.3%) patients did not sustain any postoperative complications. Thirteen patients (13/65, 20%) required revision surgery, with the predominant reason being metalwork removal due to irritation. This reflects the wide range of reported postoperative complication rates in the literature, which can be as high as 57%, with all patella fractures having a reported complication rate of 26% [13].

Although relatively uncommon, patella fractures present a significant challenge in orthopedic trauma due to the complexity of knee biomechanics and the necessity of restoring the extensor mechanism to achieve optimal function. This study evaluated outcomes of 65 patients undergoing surgical management of patella fractures using various fixation techniques. The most common fracture morphology in the cohort was transverse (32/65), followed by comminuted fractures (26/65), consistent with previously reported data [5].

TBW was the predominant fixation method, used in 42/65 (64.6%) surgically managed patients. However, complications were common, with metalwork irritation observed in 16/65 (24.6%). This was the leading reason for metalwork removal, occurring in 11 patients undergoing TBW. One patient required metalwork trimming, partial patellectomy, and extensor mechanism repair. These findings are consistent with the literature, where hardware-related symptoms are the leading cause of reoperation following TBW [14]. Other complications included osteoarthritis in 3/65 (4.6%), stiffness in 5/65 (7.7%), and malunion in 2/65 (3.1%), highlighting the importance of achieving stable fixation while preserving joint congruity and minimizing surgical morbidity. The development of osteoarthritis in this cohort underscores the potential for long-term joint degeneration, likely due to initial cartilage trauma or persistent incongruence [15].

Comminuted fractures are biomechanically more complex and often require alternative fixation strategies, such as cerclage wiring, fiber tape, or partial excision with tendon repair. These patterns demonstrated higher complication rates, including non-union, anterior knee pain, and infection [13].

Patients managed conservatively had a significantly shorter average recovery time of 2.69 months compared to 7.43 months in those managed surgically. This likely reflects that conservatively treated fractures were less severe and not associated with the physiological stress of surgery.

Patella fractures generally demonstrate a high rate of recovery to pre-injury function; however, research on optimal recovery time following surgical fixation remains limited [16]. Standardized recovery protocols suggest a return to preoperative function within three to six months [3,19] but published evidence supporting this is sparse, and recovery can vary depending on comorbidities, fracture pattern, operative fixation method, and postoperative physiotherapy [10]. Subjective preoperative function, particularly in elderly patients, can also affect these outcomes.

Pesch et al. examined recovery times in patients returning to sporting activity. In their cohort, 90.3% returned to sporting activity, but only 51.6% achieved performance levels similar to pre-injury, with a mean recovery time of 7 ± 3.9 months [17]. This differs from the three- to six-month expectation in hospital literature and may reflect the study population being physiologically fitter due to sports participation. Overall, this highlights the need for further research into postoperative recovery specific to patella fractures and fixation methods.

In this study, no appreciable difference was observed in average recovery time between patients operated on within one day (7.56 months) and those operated on two days or later (7.36 months). Literature on the optimal timing of surgery and its impact on postoperative recovery is minimal. Surgical timing is generally guided by trauma protocols, considering factors such as open versus closed fractures, injury energy, patient optimization, and surgeon availability [18]. Despite these factors, no significant difference in recovery was observed in our cohort.

Star plate and screw fixations were used less frequently but were associated with high complication rates, including stiffness and muscle weakness. These methods may be appropriate for specific fracture orientations, but their limited use and relatively poorer outcomes suggest selective application, with careful consideration of patient and fracture characteristics.

The choice of surgical technique must be individualized. While TBW remains effective for transverse fractures, its limitations are evident in oblique or comminuted patterns. In such cases, modern fixation methods, such as cannulated screws with fiber tape or low-profile plates, may provide improved outcomes, although they also carry inherent risks [19,20]. Our findings support the growing evidence emphasizing the importance of minimizing hardware prominence and optimizing postoperative rehabilitation to reduce stiffness and enhance functional recovery [21].

A notable finding was the occurrence of systemic complications, including one case of pulmonary embolism (1/65) in the TBW cohort, highlighting the need for comprehensive perioperative assessment and thromboprophylaxis.

Limitations

This study was a single-center retrospective analysis spanning 16 years, resulting in a relatively small sample size, which limited comprehensive statistical analyses. Being a single-center study, implant choice may have been biased, potentially affecting outcomes. The study focused on surgical outcomes and did not include patient-reported outcomes, which could have captured subjective recovery experiences.

## Conclusions

This study reinforces that while surgical management of patella fractures can successfully restore joint function, complication and revision rates remain significant. Transverse and comminuted fractures were the most common patterns requiring surgery, consistent with the literature. Although TBW was the most frequently used fixation method, it was associated with a high complication and reoperation rate, predominantly due to metalwork irritation. Individualized surgical technique and postoperative care are crucial to optimize outcomes. The limited data on expected recovery times and factors influencing recovery highlight areas for further research.
